# Elevational and seasonal patterns of butterflies and hawkmoths in plant-pollinator networks in tropical rainforests of Mount Cameroon

**DOI:** 10.1038/s41598-021-89012-x

**Published:** 2021-05-06

**Authors:** Jan E. J. Mertens, Lucas Brisson, Štěpán Janeček, Yannick Klomberg, Vincent Maicher, Szabolcs Sáfián, Sylvain Delabye, Pavel Potocký, Ishmeal N. Kobe, Tomasz Pyrcz, Robert Tropek

**Affiliations:** 1 Department of Ecology, Faculty of Science, Charles University, Viničná 7, 12843 Prague, Czechia; 2 Department of Biology of Organisms and Populations, Faculty of Fundamental and Applied Science, University of Poitiers, 5 rue Albert Turpain, 86000 Poitiers, France; 3 Institute of Entomology, Biology Centre, Czech Academy of Sciences, Branišovská 31, 37005 České Budějovice, Czechia; 4 Faculty of Science, University of South Bohemia, Branišovská 1760, 37005 České Budějovice, Czechia; 5 Nicholas School of the Environment, Duke University, 9 Circuit Dr., Durham, NC 27710 USA; 6 Institute of Silviculture and Forest Protection, Faculty of Forestry, University of West Hungary, Bajcsy-Zsilinszky utca 4, 9400 Sopron, Hungary; 7 Department of Invertebrate Evolution, Institute of Zoology and Biomedical Research, Jagiellonian Univeristy, Gronostajowa 9, 30-387 Kraków, Poland

**Keywords:** Ecology, Biodiversity, Community ecology, Ecological networks

## Abstract

Butterflies and moths are conspicuous flower visitors but their role in plant-pollinator interactions has rarely been quantified, especially in tropical rainforests. Moreover, we have virtually no knowledge of environmental factors affecting the role of lepidopterans in pollination networks. We videorecorded flower-visiting butterflies and hawkmoths on 212 plant species (> 26,000 recorded hrs) along the complete elevational gradient of rainforests on Mount Cameroon in dry and wet seasons. Altogether, we recorded 734 flower visits by 80 butterfly and 27 hawkmoth species, representing only ~ 4% of all flower visits. Although lepidopterans visited flowers of only a third of the plant species, they appeared to be key visitors for several plants. Lepidopterans visited flowers most frequently at mid-elevations and dry season, mirroring their local elevational patterns of diversity. Characteristics of interaction networks showed no apparent elevational or seasonal patterns, probably because of the high specialisation of all networks. Significant non-linear changes of proboscis and forewing lengths were found along elevation. A positive relationship between the lengths of proboscis of hesperiid butterflies and tube of visited flowers was detected. Differences in floral preferences were found between sphingids and butterflies, revealing the importance of nectar production, floral size and shape for sphingids, and floral colour for butterflies. The revealed trait-matching and floral preferences confirmed their potential to drive floral evolution in tropical ecosystems.

## Introduction

Recently, pollination research has shifted from detailed studies of single pollination systems to network approaches. Nevertheless, most of the complex studies of individual pollinator groups’ role in plant-pollinator networks have focused on bees or hoverflies^1,2^, whilst the other flower visitors have often been excluded or side-lined. Although some less abundant groups play important roles in pollination systems, as secondary pollinators, nectar thieves and competitors, or even as key pollinators of specialised plants^3–7^, their importance in plant-pollinator networks remains understudied, especially in tropical forests.

Compared to bees and flies, butterflies and hawkmoths represent minor pollinators in probably all terrestrial ecosystems^3,6^. Both groups are often regarded as generalised nectar feeders visiting all available nectar-rich flowers^8,9^. Even hawkmoths, considered as efficient pollinators strongly affecting floral evolution since Darwin^10^, were recently revealed as opportunistic nectar thieves of many flowers^11,12^. However, some butterflies^5,13,14^ and moths^7,9,15,16^ are key pollinators of specialised plants.

Individual lepidopteran groups differ in their morphological and behavioural adaptations to pollination. Among butterflies, papilionids, pierids, and some groups of nymphalids and hesperiids use their long proboscis to feed on nectar from deep flowers, whilst many lycaenids, riodinids, and some smaller clades within the mentioned families bear small proboscis unable to reach nectar in specialised flowers^17,18^. In moths, besides the highly specialised long-proboscid groups such as most sphingids and noctuids, adults of many groups have dysfunctional or even no proboscis^8^. Such differences hamper any attempts at quantifying the general pollination role of lepidopterans.

Plants also differ in their adaptation to butterfly or moth pollination. The pollination syndrome hypothesis^19^ expects some plants to evolve certain traits to attract the two groups. Psychophily hypothesises the adaptation for butterfly-pollination, whilst sphingophily defines hawkmoth-pollinated flowers distinguishing them from phalaenophilous plants pollinated by any other moths^8,19^. Consistently, butterflies and hawkmoths should prefer large conspicuous flowers or inflorescences^13,20^. Nocturnal hawkmoths rely comparably on colour and scent when foraging, often preferring light colours (such as white or cream) better distinguishable in the dark, and strong sweet scents^21,22^. This is in contrast with butterflies typically preferring bright flower colours, such as red or orange, above scent^23^, although sweet and fruity scents were also included into psychophily^8^. Nevertheless, the colour preference strongly varies among butterfly families and species^24,25^. Their size and proboscis length also influence flower preferences^18^. Small short-proboscid lycaenids avoid long-tubed flowers and visit small solitary flowers, long-proboscid papilionids or pierids, often larger and more energy-demanding, prefer massed nectar-rich flowers and inflorescences^17,18^. Long-proboscid hawkmoths can visit both long and short tubed flowers^9^.

Elevation and seasonality, representing various environmental and ecological gradients, influence patterns in biotic interactions^26,27^. The role, relative proportions in communities, and specific adaptations of pollinator groups may shift under differing environmental conditions, such as temperature, solar radiation, and precipitation^27,28^. Unfortunately, neither elevational nor seasonal patterns of the tropical lepidopteran role in pollination networks have been studied, except for a few case studies of individual plant species^5^. However, we can expect correlations of their role in networks with their general diversity patterns. We are not aware of any community-wide studies on characteristics of these lepidopteran-plant pollination networks in any tropical area.

Our study focuses on flower-visiting butterflies and hawkmoths, the two relatively minor groups of pollinators often overlooked in network studies, yet easily identifiable. The primary Afrotropical rainforests covering Mount Cameroon from nearly sea level to the natural timberline (ca. 2100–2300 m a.s.l. on the studied southwestern slope) offer a unique elevational gradient, with distinct dry and wet seasons. Based on rich community-wide datasets sampled along the elevational gradient and during the two seasons (Table 1), we set the following aims: (1) To evaluate the role of flower-visiting butterflies and hawkmoths in plant-pollinator networks and understand how elevation and seasonality affect their relative importance in pollination communities. (2) To analyse the elevational and seasonal changes in the structure of the pollination networks, with a specific focus on specialisation. (3) To assess butterfly and hawkmoth preferences to floral traits, as well as to test potential trait-matching between flowers and their visitors. (4) To test the potential relationship between proboscis length and specialisation of butterflies and moths in flower foraging. We hypothesise that butterflies and hawkmoths represent only a small proportion of the flower-visiting communities, although we expect their importance can be higher in lowlands and in the dry season where and when both groups are more abundant and diverse on Mount Cameroon^29,30^. We also expect higher specialisation in communities with more flower-visiting lepidopteran species, following MacArthur’s hypothesis on the positive relationship of niche breadth to species richness^31^. We expect both groups to be important pollinators of some specialised plants. We hypothesise preferences to some traits previously included in the psychophilous and sphingophilous syndromes, although we expect some predicted traits to be less important. We also expect substantial differences in the mentioned aims and hypotheses among lepidopteran families.

**Table 1:** Sites on Mount Cameroon sampled for butterflies and sphingids. ‘n.a.’ stands for data not available for particular sites.

Site				Sampled period		Number of all species		Species in pollination networks (dry/wet seasons)			
Elevation (a.s.l.)	Latitude	Longitude	Vegetation type	Checklist	Networks (dry/wet)	Butterflies	Sphingids	Butterflies	Sphingids	All plants	Visited plants
30 m	N 03.9818°	E 09.2625°	Coastal forest	Dec 2014, Jan 2015, May 2015, Oct 2017	n.a	282	5	n.a	n.a	n.a	n.a
350 m	N 04.0899°	E 09.0517°	Mosaic of primary and secondary lowland forest	Dec 2014, Apr 2015, Jan/Feb 2016	n.a	189	28	n.a	n.a	n.a	n.a
650 m	N 04.1022°	E 09.0630°	Primary lowland forest	Nov/Dec 2014, Apr 2015, Jan/Feb 2016	Jan 2018/Aug 2018	189	20	32/14	5/6	62/42	19/11
1100 m	N 04.1175°	E 09.0709°	Upland forest disturbed by elephants	Dec 2014, Jan 2015, Apr 2015, Jan/Feb 2016	Feb 2018//Sep 2018	161	8	38/7	7/4	61/32	25/12
1450 m	N 04.1443°	E 09.0717°	Submontane forest disturbed by elephants	Nov 2016, Feb 2017, Apr/May 2017	Feb 2017/Sep 2017	64	7	13/7	9/4	42/35	17/6
1850 m	N 04.1453°	E 09.0870°	Montane forest disturbed by elephants	Nov 2016, Feb 2017, Apr 2017	n.a	12	7	n.a	n.a	n.a	n.a
2200 m	N 04.1428°	E 09.1225°	Montane forest close to timberline	Nov 2016, Jan/Feb 2017, Apr 2017	Jan 2017/Aug 2017	13	3	3/0	2/1	22/28	6/2
Total						431	40	80 (69/25)	26 (19/12)	212 (144/106)	71 (54/26)

## Results

**Figure 1:**
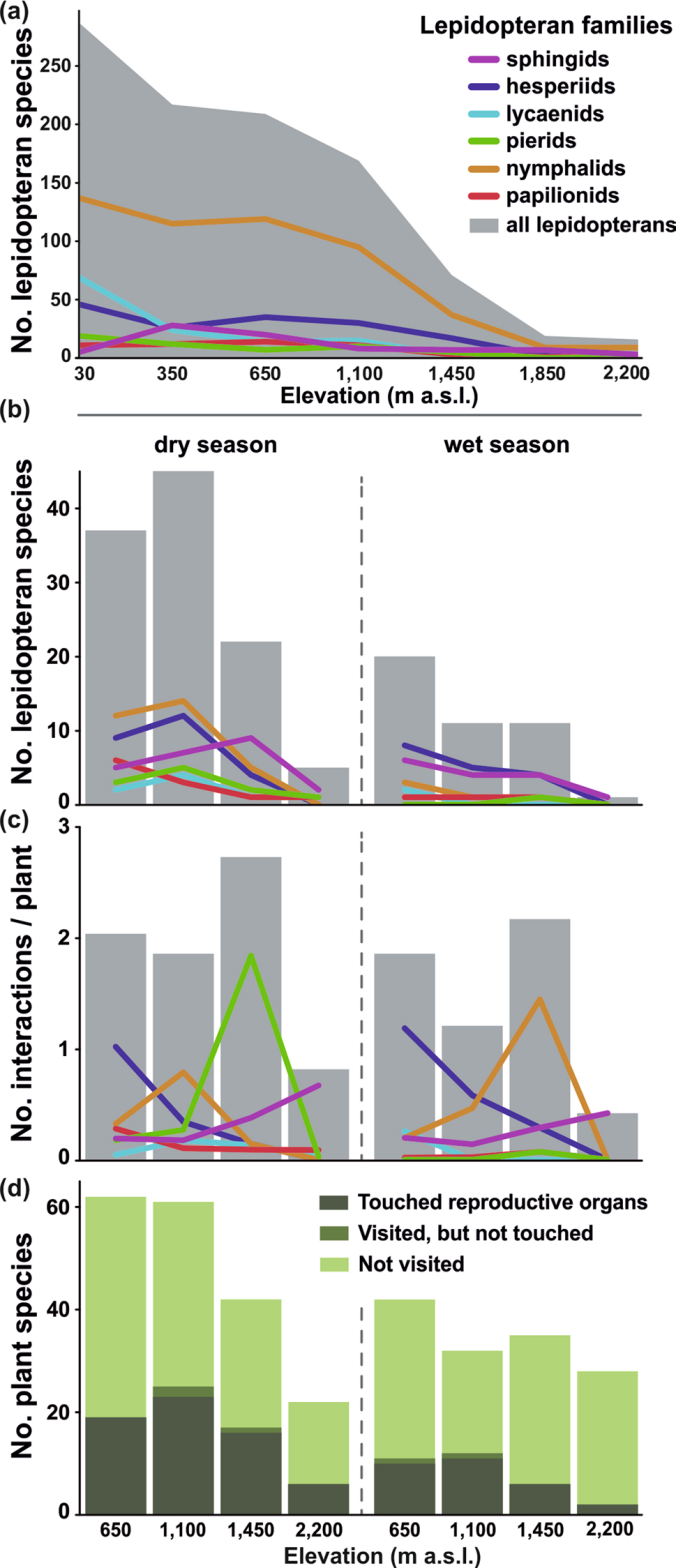
Overview of (**a**) lepidopteran species richness along the elevational gradient of Mount Cameroon, (**b**) total number of flower-visiting lepidopteran species at each elevation and season, (**c**) interaction frequency per plant and 24hrs, and (**d**) numbers of plant species whose reproductive organs were touched or untouched during lepidopteran visits, and which were not visited by any lepidopterans. Grey shading in (**a**)–(**c**) denotes the sum of all lepidopteran taxa; coloured lines represent particular families.

Altogether, our comprehensive checklist of species at seven studied elevations (from 30 to 2200 m a.s.l; Table 1) comprised from 431 butterfly and 40 sphingid species (Table 1; Fig. 1a; Supplementary Table S1). Nymphalids represented the most species across the gradient, followed by lycaenids in the lowest elevations and hesperiids at the mid-elevations (650–1450 m a.s.l.; Supplementary Table S2). Species richness of all lepidopterans and lycaenids showed a gradual decrease along elevation, whilst sphingids peaked at 350 m a.s.l. (Fig. 1a). All other butterflies showed the low plateau pattern (sensu McCain and Grytnes^32^; Fig. 1a).

Our intensive video-recording at four elevations (650, 1100, 1450, and 2200 m a.s.l.) and during dry and wet seasons (Table 1) resulted in 26,138 h (~ 2.98 years) of video footage of 1,115 individuals of 212 flowering plant species. On these video-recordings, we observed 734 individuals of 80 butterfly and 27 sphingid species visiting 71 plant species. These visits represented ~ 4% of all 18,439 flower visits recorded on the observed plants. Bees, flies, beetles, and other moths were more common flower visitors than butterflies^27^. Wasps, nectarivorous birds, and carpenter bees were more common visitors than sphingids, followed by cockroaches and mammals^27^. Still, butterflies and sphingids were among the two most common flower visitors for some plant species (Supplementary Table S3), such as *Scadoxus cinnabarinus* (Amaryllidaceae), *Distephanus biafrae* and *Melanthera scandens* (both Asteraceae), and *Cordia aurantiaca* (Boraginaceae) for butterflies; and *Anthocleista scandens* (Gentianaceae) for sphingids. From these, 700 lepidopteran visitors touched the plant reproductive organs (see Table 1 and Supplementary Table S4 for a taxonomic and spatiotemporal overview). Due to the small difference between ‘pollinators’ and ‘all visitors’ (700 and 734 interactions, respectively; Fig. 1d), we only report analyses of all interactions, i.e. the visitors’ point of view.

We recorded the highest species richness of both lepidopteran flower visitors and lepidopteran-visited plants at 1100 m a.s.l. during the dry season (Table 1; Fig. 1b). Species richness of visiting lepidopterans decreased towards the higher elevations and during the wet season; at 2200 m a.s.l. during the wet season we recorded only a single sphingid species visiting two plant species (Fig. 1b). In accordance, species richness of all flowering plants decreased towards the higher elevations and in the wet season. Yet, the highest elevation was more species-rich during wet than during the dry season. Overall, lepidopterans visited a lower proportion of flowering plant species during the wet season (wet season mean = 0.204 (± 0.114) vs. dry season mean = 0.334 (± 0.054); Fig. 1d).

The visitation frequency varied among the visitor families (Fig. 1c). Hesperiids were less frequent towards the higher elevations in both seasons. Papilionids followed such a pattern in the dry season but represented a generally small proportion of visitors in the wet season. Lycaenids were generally uncommon flower visitors with only small spatiotemporal differences. Pierids expressed a peak in frequency at 1450 m a.s.l. during the dry season (driven by *Mylothris* cf. *hilara* frequently visiting *Distephanus biafrae*). Nymphalids expressed a similar peak at 1450 m a.s.l. during the wet season (driven by *Vanessula milca* visiting *Melanthera scandens*). Finally, sphingids visited flowers more frequently towards the higher elevations in both seasons (Fig. 1c).

We found no apparent general pattern in turnover of flower-visiting lepidopterans and lepidopteran-visited plants among the studied elevations and seasons (Supplementary Fig. S1). The higher elevations shared fewer plant species with the lower elevations as well as between each other. The visitor community shared most species between 1100 and 1450 m, followed by 1450 m and 2200 m a.s.l. (Supplementary Fig. S1).

**Figure 2:**
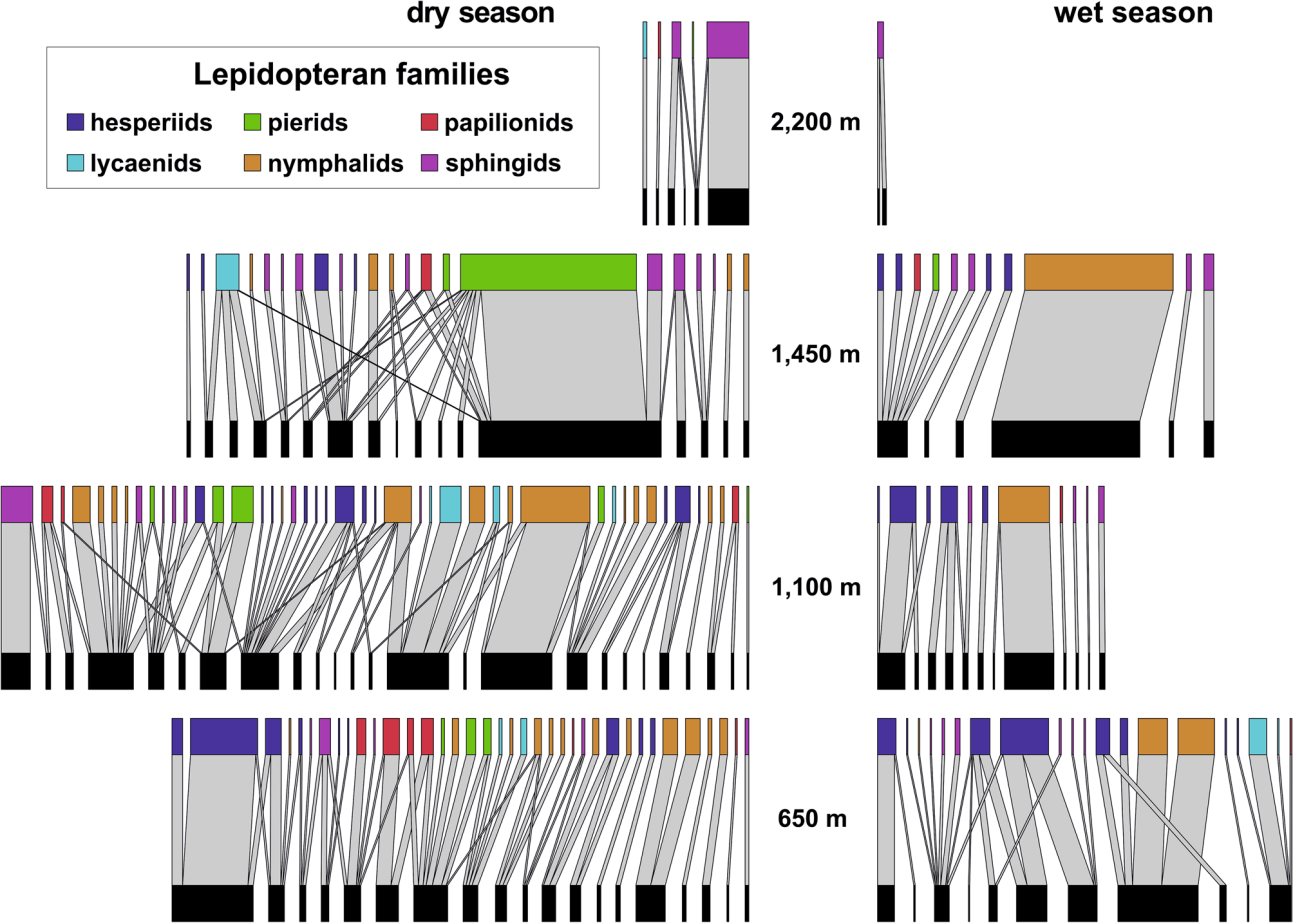
Bipartite networks of plant-lepidopteran interactions along the elevational gradient of Mount Cameroon. The upper nodes visualise flower-visiting lepidopteran species, distinguished by colour for families, whilst the lower nodes represent lepidopteran-visited plant species. The total width of each network approximates their relative size, corrected for the sampling effort (visitation frequency per 24hrs). The width of individual links (light grey) represents the relative frequency of interactions between visiting lepidopterans and visited plants within each network.

**Figure 3:**
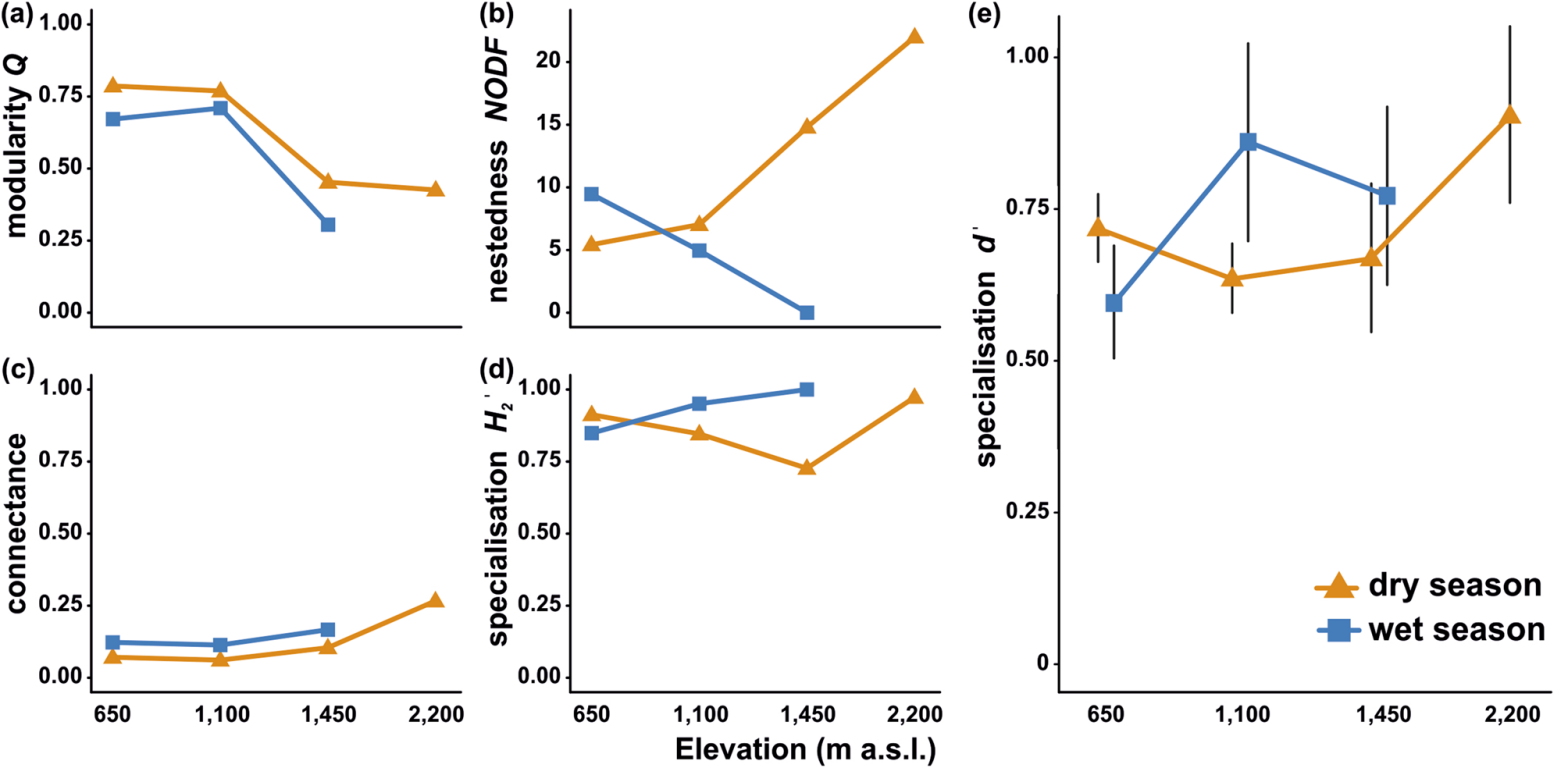
Metrics of plant-lepidopteran networks on Mount Cameroon, comparatively for each elevation and season. The symbols depict arithmetic means in all plots, whilst error bars in (e) represent 95% confidence intervals.

The reconstructed interaction networks among flowering plants and visiting lepidopterans (hereafter simplified to *plant-lepidopteran networks* or *networks*) decreased in size towards the higher elevations and the wet season, although the generally largest network was recorded at 1100 m a.s.l. in the dry season (Fig. 2). The trends in the network characteristics were minor or none, except NODF nestedness. Network connectance slightly increased along the elevational gradient and remained similar between seasons. Q modularity slightly decreased towards the higher elevations and during the wet season. NODF nestedness increased along elevation during the dry season and showed an opposite trend during the wet season. H_2_′ specialisation slightly increased along elevation during the wet season, whilst no pattern was observed during the dry season (Fig. 3a–e).

The studied lepidopteran families did not significantly differ in *d’* specialisation (LMM, *F* = 0.865, *p* = 0.508), nor there was any significant difference in *d’* specialisation between sphingids and all butterflies (LMM, *F* = 0.287, *p* = 0.593). Model comparisons of the effects of elevation, season, and their interaction showed that the interaction effect of both factors is the most plausible descriptor of the observed patterns in *d’* specialisation (Fig. 3e; Table 2).

**Table 2:** Comparison of the effects of season, elevation, and their interaction on *d’* specialisation of flower-visiting lepidopterans on Mount Cameroon. LMM with the lepidopteran families as random-effect variable were applied; models with *ΔAICc* ≤ 2 were considered comparable.

Model	Residual df	Residual deviance	ΔAICc	Weight	R^2^ adj
Season	149	7.00	11.1	0	0
Elevation	147	6.77	10.3	0.01	0.017
Season × elevation	**144**	**6.05**	**0**	**0.99**	**0.102**

### Patterns in lepidopteran morphological traits

**Figure 4:**
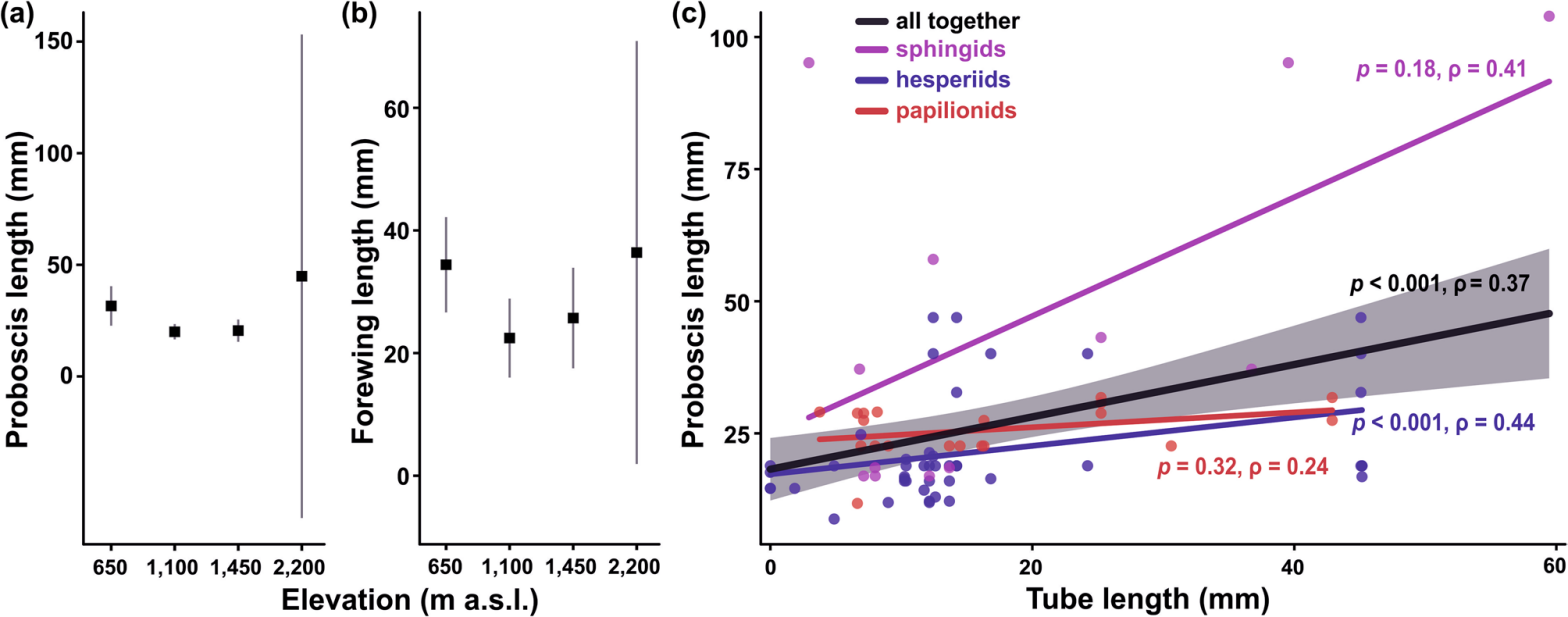
(**a**) Proboscis and (**b**) forewing lengths of flower-visiting lepidopterans on Mount Cameroon. Mean values and 95% confidence intervals are visualised. (**c**) Spearman correlations of lepidopteran proboscis length and corolla tube length of lepidopteran-visited plants. Each data point represents an interaction between a plant species and a lepidopteran species. The black line visualises correlation of all data (with grey shaded confidence intervals), whilst the coloured lines visualise correlations of individual lepidopteran families.

Lengths of proboscis and forewing, measured in hesperiids, papilionids, and sphingids, differed significantly among the families (proboscis length: LMM, *χ*^*2*^ = 12.15, *df* = 2, *p* = 0.002; forewing length: LMM, *χ*^*2*^ = 31.95, *df* = 2, *p* < 0.001). Sphingids had on average the longest proboscides, followed by papilionids and hesperiids (Supplementary Table S5). Papilionids had on average the longest forewings, followed by sphingids and hesperiids (Supplementary Table S5). None of the three families showed any significant patterns in their proboscis or forewing lengths along the elevational gradient (Supplementary Table S6). However, when analysing only the flower-visiting species from our video-recordings, elevation became the most plausible descriptor of the non-linear patterns in their proboscis and forewing lengths (Table 3; Fig. 4a,b). Both traits were longest in the lowest elevation, whilst both mid-elevations were comparable. The highest elevation showed too large variability in both traits (caused by the low number of measured lepidopterans) for any reasonable interpretations. Even though only three species were measured at the highest elevation, omitting them had no substantial effect on the model parsimony (Supplementary Table S7). We found a significantly positive correlation between lepidopteran proboscis length and corolla tube length of lepidopteran-visited flowers (Fig. 4c). However, from the three lepidopteran families, the observed relationship was only significant for hesperiids when analysed separately (Fig. 4c). Neither proboscis nor forewing lengths had any significant effect on *d’* specialisation (proboscis length: Spearman, *p* = 0.38, *ρ* = 0.16; forewing length: Spearman, *p* = 0.63, *ρ* = 0.086).

**Table 3:** Linear model comparison of the individual effects of season and elevation, and their interaction, on proboscis and forewing length of lepidopterans on Mount Cameroon. ‘*res. df*’ and ‘*res. dev.*’ represent the residual’s degrees of freedom and deviance, respectively.

Model	Res. df	Res. dev	ΔAICc	Weight	R^2^ adj
**Proboscis length**					
Season	51	73.069	3.2	0.16	0.007
Elevation	**49**	**64.940**	**0**	**0.78**	**0.114**
Season × elevation	45	58.864	5.2	0.06	0.180
**Forewing length**					
Season	52	15.631	9.4	0.01	0
Elevation	**50**	**1.27**6	**0**	**0.88**	**0.062**
Season × elevation	46	− 5.802	4.1	0.11	0.090

### Lepidopteran preferences to floral traits

**Figure 5:**
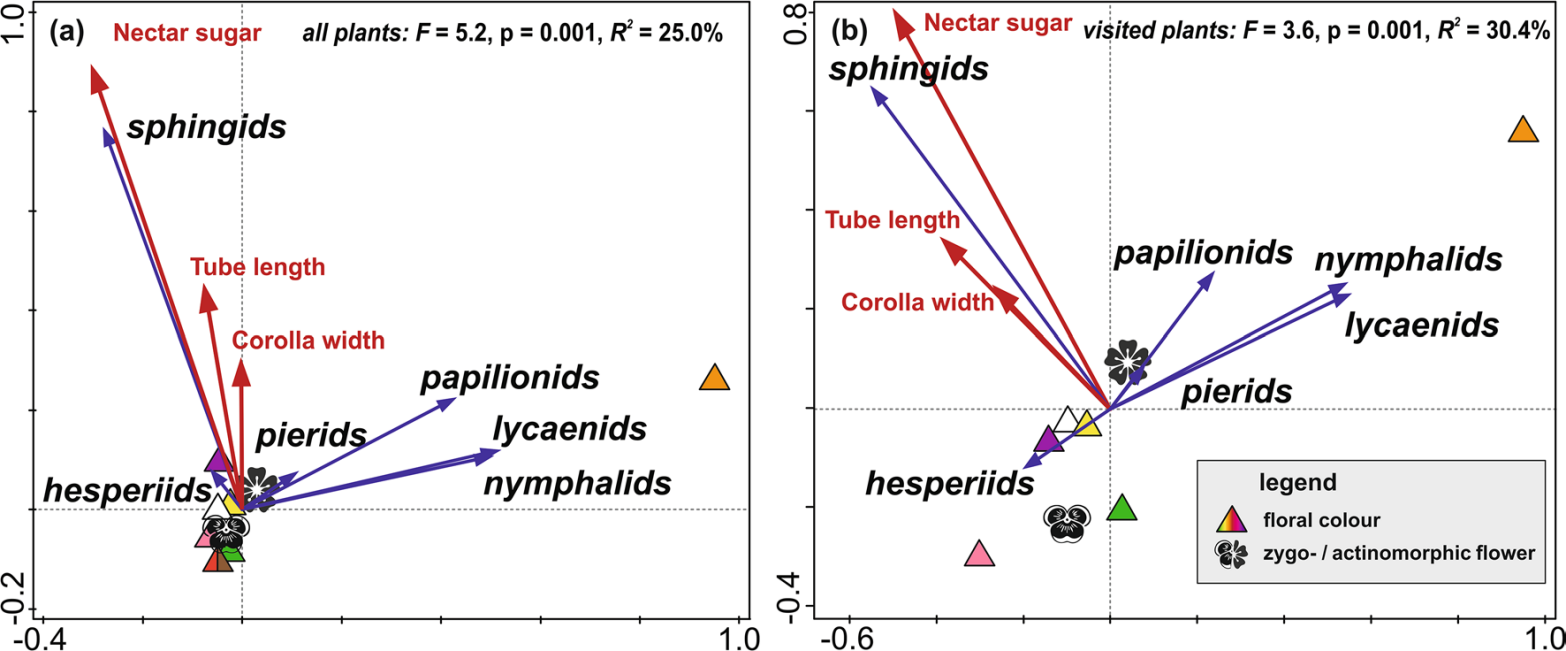
Redundancy analyses (RDA) revealing significant preferences of butterfly and sphingid families (represented by blue arrows) to floral traits (represented by red arrows and various symbols) on Mount Cameroon. The two RDA models were run for (**a**) all flowering plant species, and (**b**) the plant species visited by butterflies or moths.

Finally, we analysed preferences of individual lepidopteran families to specific floral traits by multivariate RDA ordinations^33^. Flower-visiting lepidopterans showed significant preferences towards certain floral traits (Fig. 5). With the dataset of all flowering plants, the selected floral traits explained 25% of the variability in the visitation frequency (Fig. 5a). The focal families formed three relatively distinct groups. Sphingids preferred sugar-rich, larger and deeper flowers of purple colour. Papilionids, lycaenids, and nymphalids preferred orange flowers, whilst hesperiids and pierids did not express any apparent preferences to floral traits. These preferences were mostly consistent with the analysis including only the Lepidoptera-visited flowers (Fig. 5b), although hesperiids preferred pink actinomorphic flowers.

## Discussion

### Importance of butterflies and sphingids as pollinators

On Mount Cameroon, butterflies and sphingids did not represent the most important pollinators in the studied tropical rainforests, as they together made up ~ 4% of the flower-visiting community. Their numbers were dwarfed by flower-visiting bees, flies, and beetles, representing 44.10%, 25.71% and 11.83% of visits of all recorded plants, respectively^27^. The relative importance of lepidopterans in our uniquely comprehensive Afrotropical networks was even lower than in several partial networks from tropical forests of South-East Asia (e.g.^34,35^) and the Neotropics (e.g.^36,37^). In all these studies, flower visitation by lepidopterans was considerable (between 10 and 20% of all pollinators, although nocturnal visitors were ignored which could have increased the relative importance of diurnal lepidopterans), although incomparable to bees (between 40 and 55%). We are not aware of any similar study from Afrotropical forests.

Even though butterflies and sphingids visited about a third of all flowering plants in the study area, only a few plant species seemed to be primarily pollinated by these groups. Butterflies were the most common visitors of a single plant species, *Scadoxus cinnabarinus*, already known to be butterfly-pollinated^5^. In only a few other plant species, butterflies ranked high among all visiting groups (Supplementary Table S3). Sphingids were not the most common visitors of any recorded plant species, but they ranked second among the groups visiting *Anthocleista scandens*. Although plants within this genus have been reported as potentially pollinated by moths or sunbirds^38^, its widely open typical chiropterophilous flowers^39^ do not morphologically fit to sphingophily. However, several other plants commonly visited by butterflies (e.g. *Aframomum* spp. and *Cordia aurantiaca*) and sphingids (e.g. *Mussaenda tenuiflora* and *Clerodendrum silvanum*) in our study offer morphologically specialised flowers fitting the lepidoptera-related pollination syndromes^8^. Their efficient pollination by lepidopterans can be expected from other studies of relative or similar plant species from other areas^40,41^. Several other plants commonly visited by the studied lepidopterans offered rather morphologically generalised inflorescences (e.g. *Distephanus biafrae*, *Melanthera scandens*, and *Crassocephalum montuosum*; all Asteraceae). Plants with such inflorescences have sometimes been reported as pollinated by butterflies or moths, although they were visited by rich pollinator communities and apparently did not only rely on lepidopterans^42,43^. Altogether, only a few plant species in our study seemed to depend on pollination by butterflies or sphingids based on the combination of their flowers’ morphology and visitation frequency. In conclusion, butterflies and sphingids seem to be relatively less relevant pollinators in Afrotropical forests.

### Seasonal patterns of lepidopteran pollination

Pollination networks of butterflies and sphingids strongly differed between the studied seasons on Mount Cameroon. This was surely influenced by a plethora of factors affecting communities of both flower-visiting lepidopterans and flowering plants. The very high inter-seasonal turnover of both butterfly and moth species composition, as well as changes in species richness and abundance, have already been reported in detail from Mount Cameroon^29^, as well as from other Afrotropical rainforests (e.g.^44^). Together with the confirmation of the high species turnover between dry and wet seasons (Supplementary Fig. S1), this study also revealed seasonal changes in lepidopteran behaviour and networks of their pollination interactions. The general decline of flower-visiting butterflies is most probably connected to the local extreme precipitation during the wet season. Adult butterflies and sphingids, like other large-winged insects, avoid the extreme rainfall on Mount Cameroon^30^. Simultaneously, the strong rains and related humidity also affect availability of nectar and its concentration^45^. The strong seasonality affects the flowering plant communities as well. Whereas many trees, usually offering large amounts of generally accessible nectar, flower during the dry season, herbs and shrubs flowering in the wet season are not able to substitute this nectar production. Our unpublished suggestions were supported by the relatively high turnover of flowering plants between the two seasons (Supplementary Fig. S1). Moreover, herbs flowering in the harsh conditions during the wet season on Mount Cameroon are often adapted to pollination by sunbirds^46,47^. These could be both causes and consequences of the generally lower diversity and abundance of nectarivorous lepidopterans during the wet season.

### Elevational patterns of lepidopteran pollination

Species richness of butterflies and sphingids showed the ‘low-elevation plateau with a mid-peak’ (sensu McCain and Grytnes^32^) in accordance with numerous studies of tropical lepidopterans^30,48^. The flower visitation frequencies of butterflies mirrored this result, whilst the importance of sphingids in pollination networks, both absolute and relative, increased along elevation. Because we are not aware of any studies on plant-lepidopteran pollination networks along any tropical elevational gradient for comparison, we can hardly speculate if the revealed patterns can be general. However, a study of the *Scadoxus cinnabarinus* pollination system showed peaking diversity and abundance of flower-visiting butterflies at mid-elevations of the plant range^5^. Besides numerous factors responsible for the generally high diversity at mid-elevations in many insect groups^48,49^, the patterns of plant-lepidoptera networks can be discussed in relation to the floral resources for nectarivorous butterflies and sphingids. Although we have no detailed data on the abundance of floral resources along the studied elevational gradient, the local diversity of trees linearly decreased along the gradient^50^. However, it remains questionable whether all flowering plants follow this pattern. The opposite trends of sphingid species richness and their importance in networks may be related to the dominance of a few highly mobile species among flower-visiting sphingids in all networks. Whilst their elevational diversity pattern was driven by numerous species with restricted elevational ranges on Mount Cameroon^30^, numerous identified sphingids in our networks were opportunistic long-distance vagrants (Supplementary Table S1). Considering the generally small sizes of the plant-sphingid networks and the relatively smaller elevational variability, the revealed patterns could be caused by more or less random visitation by these mobile generalists.

The prevailingly non-apparent trends in the plant-lepidopteran network characteristics can be surprising because several other studies of pollination networks along elevation revealed strong patterns, although inconsistent among the studied areas and groups^1,51^. Nevertheless, we are not aware of any similar study on pollination by butterflies or sphingids along any tropical elevational gradient for comparison. Therefore, we hypothesise that lepidopteran species are surprisingly specialised for visited flowers, as visible in our relatively less connected and highly specialised networks, and in the preferences for distinct floral traits. These preferences only partly change with environmental conditions^27^. However, as discussed above, only a minor portion of the visited flowers are specialised for lepidopteran pollination. Nevertheless, only more studies of plant-lepidopteran networks from other tropical areas can challenge such hypotheses. We admit that for the only characteristic with a strong trend, nestedness, we do not have any apparent explanation, especially because the trends strongly differed inter-seasonally.

### Traits in plant-lepidopterans networks

We found no evidence that longer proboscides or forewings of butterflies and sphingids elicited differences in the flower visitation behaviour, apart from the correlation between lengths of proboscis and floral tube of flowers visited by hesperiids. No proboscis-tube relationship has been found in other studies (e.g.^9,12,52^), although some studies found larger butterflies visiting larger flowers as well^17,18^. Nevertheless, although preferring flowers with longer tubes, the long-proboscid lepidopterans were not more specialised with respect to their food-niche breadth (relative number of visited plant species) in our study. Therefore, we assume that even these morphologically specialised lepidopteran species were looking for any available resources in longer or deeper flowers which are more likely to be unreachable by other floral visitors, rather than being specialised for a few co-evolved plant species (cf.^9,12^).

Although we found no significant patterns among the morphological traits of lepidopterans along elevation when analysing all captured butterflies and sphingids, the lengths of forewing and proboscis showed significant non-linear patterns when the analyses were restricted to the flower-visiting species. We expect that such patterns can be obscured by other mechanisms when analysing the complete lepidopteran community, including species with adults feeding on other resources than flowers. The increase of lepidopteran size towards the higher elevations was repeatedly reported and mostly explained by the need of a larger surface for basking in colder environments^53^. Nevertheless, the relatively larger bodies of lepidopterans in the lowest elevation seems surprising and difficult to explain.

Generally, preferences of butterflies and sphingids to the visited flowers were driven by floral colour, size, and nectar-sugar production in our study. This corroborates numerous other studies^8,18^. Moreover, the floral preferences neatly separated nocturnal sphingids from diurnal butterflies, as proposed by the pollination syndrome hypothesis^8^. Concurrent with other studies^9,54^, sphingids preferred longer and nectar-rich flowers on Mount Cameroon. Opposite to the sphingophilous syndrome^8^, they did not seem to prefer white flowers. Papilionids, nymphalids and pierids preferred orange flowers, the other floral traits were much less relevant. Strong preferences to floral colours have already been shown for butterflies, although inconsistently for individual families and species^23,24^; orange flowers were hypothesised as typical for psychophilous plants^8^. Pierids and hesperiids expressed little to no preferences to any floral traits in this study. Such differences among butterfly families have already been observed^24,25^. Our detailed taxon-specific approach uncovered some of the limitations of the pollination syndrome hypothesis, especially that different traits can differ in their importance among particular syndromes, and that even individual subgroups of the single pollinator group can differ in their preferences^27,55^.

## Methods

### Study area

Mount Cameroon (4095 m a.s.l.) is an active volcano in the Southwest Region, Cameroon, West/Central Africa. Primary tropical rainforests cover its southwestern flanks, where the study was performed, from lowlands (above human encroachment at ca. 300 m a.s.l.) up to the natural timberline (ca. 2200 m a.s.l.). As the mountain is located within the ‘Guinean forests of West Africa’ biodiversity hotspot, it holds an extraordinarily high biodiversity of numerous taxa, including butterflies^56^, hawkmoths^57^, and plants^58^. The mountain is one of the wettest places in the world and experiences distinct dry (December–February) and wet seasons (June–September)^29,30^. The Atlantic Ocean-facing southwestern lowlands receive large amounts of rainfall (> 12,000 mm annually), most of which occurs during the wet season (> 2500 mm monthly), and rarely any rain during the dry season^30^. To characterise changes in plant-pollinator interactions along elevation and season, we studied four sites on the southwestern slope at 650, 1100, 1450, and 2200 m a.s.l. The butterfly and hawkmoth species richness data includes additional sampling sites at 30, 350, and 1850 m a.s.l. (Table 1). For more details on the study sites, see Maicher et al.^30^.

### Study groups and their biodiversity patterns

This study focused on butterflies (Lepidoptera: Papilionoidea) and hawkmoths (Lepidoptera: Sphingidae; referred to as sphingids in the manuscript). For part of the analyses, butterflies were split up into their families (Hesperiidae, Papilionidae, Pieridae, Lycaenidae, and Nymphalidae; referred to as hesperiids, papilionids, pierids, lycaenids, and nymphalids in the manuscript). All butterflies and sphingids were represented in the *flower visitation* and *floral preferences* parts of the study. However, the effects of elevation and season on visitor size and proboscis length were analysed for papilionids, hesperiids, and sphingids only, because the other groups’ traits were not measured in the field. We actively inventoried butterflies and sphingids along the complete elevation of Mt. Cameroon (i.e. at seven elevations from 30 to 2200 m a.s.l.), using the checklist approach. For this purpose, we applied intensive hand-catching (our unpublished data) and bait-trapping (using fermented mashed bananas; data from Maicher et al.^30^) for butterflies, whereas we used standardised light-attraction for sphingids (data from Maicher et al.^30^). These data were further supplemented by a few additional species found only in the video recordings described below.

### Flower visitation

We recorded flower-visiting lepidopterans at four elevations (650, 1150, 1450, and 2200 m a.s.l.), along six transects (200 × 10 m) per elevation established to characterise the local vegetation heterogeneity^27^. Along those transects, we recorded flower visitors of all plant species flowering during our fieldwork (two weeks during dry and wet seasons at each elevation; Table 1) using security cameras with IR night-vision (Vivotek IB8367RT). We positioned the cameras 0.5–1.5 m from the flowers or inflorescences and camouflaged their surfaces. We recorded flowering plants at all vegetation layers from understorey to canopy, using ladders and climbing ropes to reach higher strata. Five individuals of each plant species were recorded, each for a 24-h session. The individual replicates were separated in space (different transects) and time (different days). During the first week, we added any plant species that had been flowering. The second week served towards completing the necessary five replicates and no more species were added to the study. Whenever insufficient individuals flowered along the transects, we searched the adjoining area.

We observed all flower visitors from the video recordings either through semi-autonomic motion detection with Motion Meerkat 2.0.5^59^ when conditions allowed, or manually through sped-up playback. We identified all butterflies and sphingids using diverse available literature and the large reference collection we have established during our work on Mt. Cameroon since 2014, and in the Gulf of Guinea Highlands since 2007. Most of the visitors were identified to species, whilst 7 morphospecies were established for butterflies and 18 for sphingids (Supplementary Table S1). In most of these cases (except 8 sphingid morphospecies), we were able to identify visiting lepidopteran to genus and to distinguish the morphospecies from all other species or morphospecies in our dataset, despite that we failed to assign them to any species name. For each visiting Lepidoptera, we determined whether they touched the plant’s reproductive organs (anthers, stigmata, or both) to distinguish potential pollinators from other visitors.

The recorded interactions were used to reconstruct individual plant-lepidopteran networks for each elevation and season (i.e. eight networks). We used visitation frequency (i.e. number of interactions of each species per plant species during 24 h) in each of the eight networks. This controls for differences in total recording time between plant species in the few cases we failed to find enough replicates, or the recordings were shorter because of dying flowers or technical failures.

To visualise and characterise the eight plant-lepidopteran networks, we used the *bipartite* package^60^ in R 3.5.3^61^. We quantified network *connectance*^62^, network-level *H*_*2*_*′ specialisation*^63^, *Q modularity*^64^, and *NODF nestedness*^65^. We calculated each metric firstly including all floral visitors, and secondly only with the subset of visitors touching the plants’ reproductive organs. Because of the highly limited number of replicates (each combination of elevation and season was characterised by a single network), any possible elevational and seasonal patterns of the network characteristics were checked by a direct comparison of values, i.e. without any statistics. Finally, we calculated *d’ specialisation*^63^ of each lepidopteran species in each network. The relationship of the species-level specialisation of lepidopterans to elevation and season was analysed by a linear mixed-effect model (LMM) with specialisation of individual lepidopteran species in particular networks as a continuous response variable, and with elevation and season as categorical explanatory variables. Individual lepidopteran families were included as a categorical random-effect factor to correct for the inter-family variability.

### Relationship between floral and lepidopteran traits

We measured five floral traits of 174 plant species included in the plant-pollinator networks: *symmetry* (actino- or zygomorphic), prevailing *floral colour*, *corolla width*, floral *tube length* (distance from the flower opening to its base, or tip of the spur when present), and *nectar sugar* (total mass of sugars produced by a flower during 24 h; the sampling protocol followed Bartoš et al.^66^.

We measured eight morphometric traits of 1,665 specimens of 130 lepidopteran species (75 hesperiids, 15 papilionids, and 40 sphingids) collected during the project. Directly in the field, we *weighed* fresh specimens and cut their proboscides for later measurement. The collected specimens were mounted and photographed at the Nature Education Centre, Jagiellonian University, Krakow. On these photographs, we measured *forewing length* and *width*, *body length*, and *thorax width*, *lengths of fore*-, *mid*- and *hindleg*, and *proboscis* (Supplementary Fig. S2) in ImageJ2^67^, with hundredths of mm accuracy. We assessed the lepidopteran trait collinearity by multiple regression, and selected the proboscis (independent of most other traits) and forewing lengths (correlated with the other traits and therefore being a useful proxy for the specimen size) for analyses (Supplementary Table S8).

We analysed patterns of the proboscis and forewing lengths in communities of all lepidopterans measured at different elevations by LMM. The average trait values per species were used as response variable (log-transformed, as the data showed a lognormal distribution), elevation as categorical fixed-effect variable, and lepidopteran families as random-effect variable to correct for inter-family variability. Consequently, we analysed elevational and seasonal (fixed-effect variables) differences in the proboscis or forewing lengths (response variables) in flower-visiting lepidopteran species only. This dataset involved 34 measured lepidopteran species recorded during flower visits (19 hesperiids, 7 papilionids, 8 sphingids). In both analyses, we applied AICc (AIC corrected for small samples^68^) to select the most plausible models. Due to the high variability in sample sizes, no post-hoc tests were performed. Finally, we tested the correlations between the proboscis and forewing length of lepidopterans and their d’ specialisation using Spearman’s rank coefficients; after Bonferroni correction for multiple comparisons, *p* < 0.0125 were considered as a significant value in these analyses (α).

### Floral preferences

We assessed how the floral preferences to particular floral traits differ among the six focal lepidopteran families by ordination analyses in Canoco 5^69^. All five measured flower traits served as explanatory variables towards the visitation frequencies by lepidopteran species (response variable). Based on the gradient lengths, two RDA models were selected and tested using 999 Monte Carlo permutations^33^. Firstly, to assess lepidopteran preferences within the whole local community of flowering plants, we included all plant species for which we measured the traits (n = 173). Subsequently, we analysed lepidopteran preferences only among the visited plant traits (n = 63). Finally, we tested whether lepidopterans with longer proboscides visit flowers with longer corolla tubes by correlating the average proboscis length of the 34 measured lepidopteran visitors with the corolla tube length of the visited plant species using Spearman’s rank coefficient.

## Supplementary Information

Supplementary Information

## Data Availability

Data available via the Zenodo repository (*doi: 10.5281/zenodo.4711162*).
