# Supplementary material for: Elevational and seasonal patterns of butterflies and hawkmoths in plant-pollinator networks in tropical rainforests of Mount Cameroon
Source: Sci Rep. 2021 May 6;11:9710. doi: 10.1038/s41598-021-89012-x (PMC8102585; doi:10.1038/s41598-021-89012-x)
Supplement: Supplementary file 1 — Supplementary Information [file 41598_2021_89012_MOESM1_ESM.pdf]

SUPPLEMENTARY INFORMATION

Elevational and seasonal patterns of butterflies and hawkmoths in plant-pollinator networks in tropical rainforests of Mount Cameroon.

Jan E.J. Mertens, Lucas Brisson, Štěpán Janeček, Yannick Klomberg, Vincent Maicher, Szabolcs Sáfián, Sylvain Delabye, Pavel Potocký, Ishmeal N. Kobe, Tomasz Pyrcz and Robert Tropek.

Supplementary Figure S1. Turnover of lepidopteran and plant species in the studied plant-lepidoptera pollination networks on Mount Cameroon, visualised as proportions of the shared butterfly and sphingid visitor species and visited plant species among particular elevations and seasons.

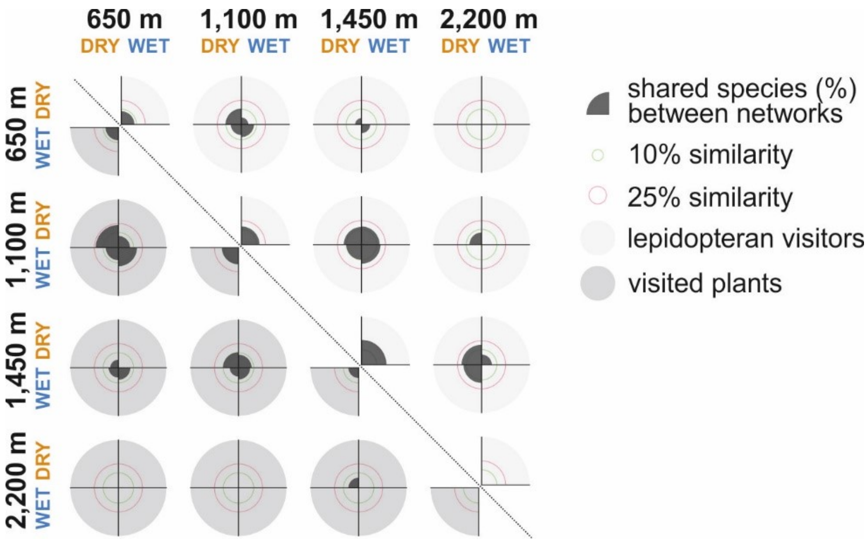

Supplementary Figure S2. Illustration of selected measured traits (as visualised by the red lines) of flower-visiting butterflies and sphingids on Mount Cameroon. (A) *Forewing length*: from the wing base to the wingtip (defined as the point where the tangent of a wing edge is perpendicular to the measure line). (B) *Forewing width*: perpendicular to the forewing length measure line and positioned so that the tangent of the outer wing margin is perpendicular to the measure line. (C) *Body length*: from the top of the head (excluding mouthparts) to the end of the abdomen (excluding genital valves). (D) *Body width*: measured where forewings attach to the thorax. (E-G) *Lengths of fore-, mid-, and hindleg*, respectively: from the base of the tibia to the last tarsus (excluding tarsal claw), measured by a segmented line.

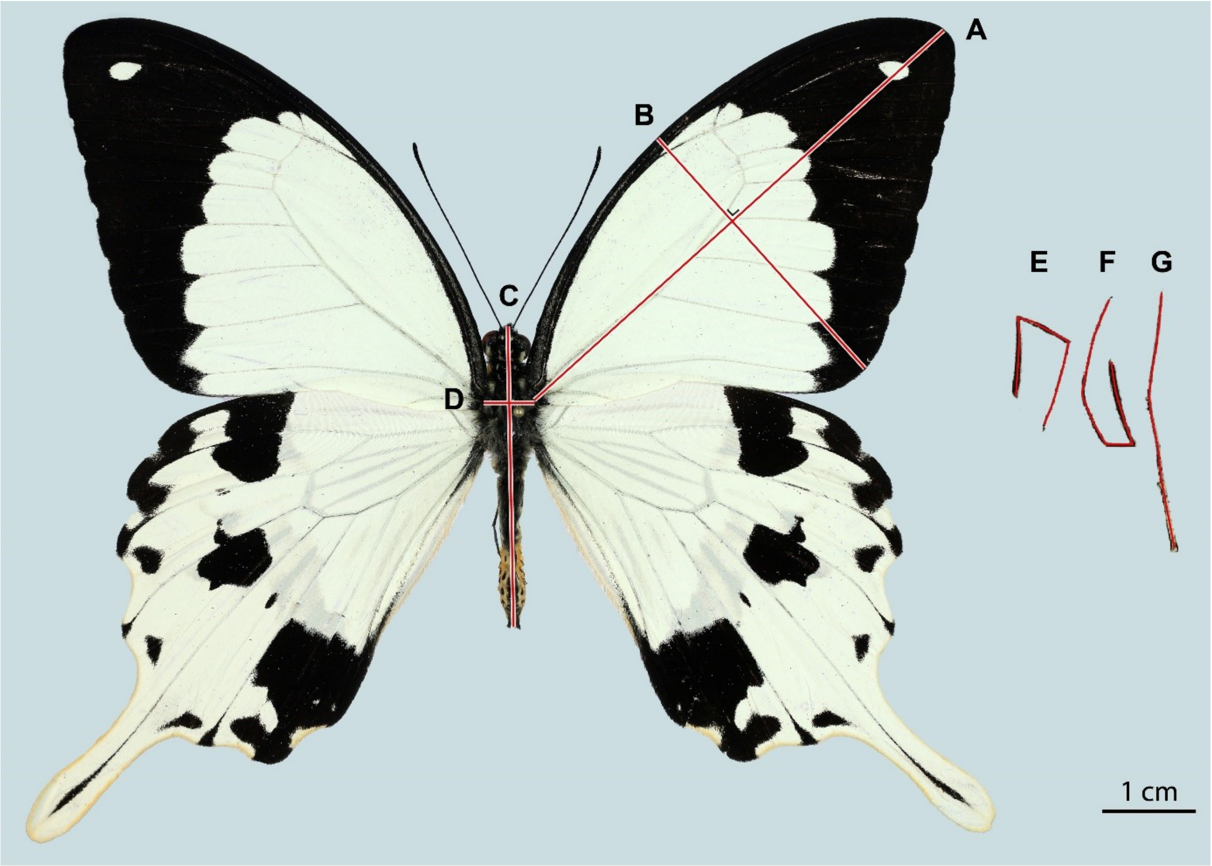

Supplementary Table S1. List of all flower-visiting butterflies and sphingids identified from the video recordings on Mount Cameroon.

Hesperiidae	***Nymphalidae***	***Papilionidae***
Acleros bibundica	*Acraea alcinoe*	*Graphium policenes*
Andronymus magma	*Acraea bonasia*	*Papilio charopus*
Andronymus sp.1	*Acraea elongate*	*Papilio cyproeofila*
Apallaga alluaudi	*Acraea epaea*	*Papilio dardanus*
Apallaga intermixtus	*Acraea judutta*	*Papilio hesperus*
Apallaga meditrina	*Acraea lycoa*	*Papilio menestheus*
Apallaga mona	*Acraea penelope*	*Papilio zenobia*
Bettonula bettoni	*Acraea pharsalus*	*Papilio zoroastres*
Celaenorrhinus dargei	*Acraea rogersi*	
Ceratrichia clara	*Acraea tellus*	**Sphingids**
Ceratrichia fako	*Acraea umbra*	*Agrius convolvuli*
Ceratrichia flava	*Amauris damocles*	*Centroctena rutherfordi*
Coeliades chalybe	*Amauris echeria*	*Hippotion osiris*
Coeliades forestan	*Bebearia tentyris*	*Hippotion* sp.1
Coeliades hanno	*Bicyclus anisops*	*Hippotion* sp.2
Coeliades libeon	*Bicyclus sciathis*	*Hippotion* sp.3
Eagris decastigma	*Cymothoe beckeri*	*Macroglossinae* sp.1
Gorgyra sp.1	*Cymothoe consanguis*	*Macroglossinae* sp.2
Leona lissa	*Cymothoe herminia*	*Macroglossinae* sp.3
Meza sp.1	*Cymothoe indamora*	*Macroglossinae* sp.4
Osmodes lux	*Cymothoe sangaris*	*Macroglossinae* sp.5
Osmodes thora	*Cymothoe weymeri*	*Macroglossinae* sp.6
Paracleros sp.1	*Euphaedra hewitsoni*	*Macroglossinae* sp.7
Pardaleodes tibullus	*Euphaedra losinga*	*Macroglossinae* sp.8
Paronymus xanthias	*Euphaedra* sp.1	*Macroglossum trochilus*
Semalea sp.1	*Euphaedra temerraria*	*Nephele accentifera*
Tagiades flesus	*Hypolimnas salmacis*	*Nephele* sp.1
	Junonia terea	*Nephele* sp.2
Lycaenidae	*Phalanta eurytis*	*Nephele* sp.3
Anthene definita	*Precis milonia*	*Nephele* sp.4
Cacyreus cf. *lingeus*	*Vanessula milca*	*Temnora iapygoides*
Euchrysops malathana		*Temnora* sp.1
Hypolycaena sp.1	***Pieridae***	*Theretra orpheus*
Neurypexina lamprocles	*Appias* cf. *sabina*	*Theretra* sp.1
Thermoniphas alberici	*Leptosia alcesta*	*Theretra* sp.2
Zizula hylax	*Leptosia* cf. *hybrida*	*Xanthopan morganii*
	Leptosia nupta	

Supplementary Table S2. Species richness of individual butterfly families recorded at each sampled elevation on Mount Cameroon; see Table 1 for species richness of sphingids, as well as for the cumulative numbers.

Elevation (m a.s.l.)	Butterfly family				
	Hesperiidae	Lycaenidae	Nymphalidae	Papilionidae	Pieridae
**30**	46	69	137	11	19
**350**	26	24	115	12	12
**650**	35	14	119	14	14
**1,100**	30	15	95	11	11
**1,450**	17	2	37	3	3
**1,850**	2	0	9	1	1
**2,200**	1	1	9	1	1

Supplementary Table S3. Plant species with most visits by butterflies (top 10) and hawkmoths (top 5) on Mount Cameroon, as well as the rank of butterflies/sphingids among all functional groups of flower visitors (Klomberg et al. 2020).

**butterflies**			
**plant family**	**plant species**	**No. visits**	**rank**
Asteraceae	*Distephanus biafrae*	136	2
Zingiberaceae	*Aframomum* sp. ‘purple’	67	3
Compositae	*Melanthera scandens*	63	2
Amaryllidaceae	*Scadoxus cinnabarinus*	39	1
Balsaminaceae	*Impatiens macroptera*	27	3
Vitaceae	*Cissus oreophylla*	24	3
Apocynaceae	*Tabernaemontana ventricosa*	22	5
Asteraceae	*Crassocephalum montuosum*	22	5
Boraginaceae	*Cordia aurantiaca*	17	2
Balsaminaceae	*Impatiens mannii*	16	3

**sphingids**			
**plant family**	**plant species**	**No. visits**	**rank**
Gentianaceae	*Anthocleista scandens*	15	2
Asteraceae	*Distephanus biafrae*	13	6
Rubiaceae	*Mussaenda tenuiflora*	11	3
Apocynaceae	*Tabernaemontana ventricosa*	8	7
Lamiaceae	*Clerodendrum silvanum*	7	4

Supplementary Table S4. Summary of butterflies and sphingids visiting flowers / touching flowers' reproductive organs at individual elevations and seasons on Mount Cameroon.

Elevation (m a.s.l.)	season	butterflies hesperiids	lycaenids	nymphalids	papilionids	pierids	sphingids	all lepidopterans
**650**	**dry**	69 / 65	3 / 3	22 / 21	19 / 19	12 / 12	13 / 13	138 / 133
**1,100**	**dry**	33 / 24	17 / 17	76 / 75	10 / 10	26 / 26	17 / 17	179 / 169
**1,450**	**dry**	11 / 2	10 / 10	11 / 11	7 / 7	141 / 137	29 / 28	209 / 195
**2,200**	**dry**	0/0	1 / 1	0/0	3 / 3	1 / 1	23 / 23	28 / 28
**650**	**wet**	60 / 57	13 / 13	10 / 10	1 / 1	0/0	10 / 10	94 / 91
**1,100**	**wet**	25 / 23	0/0	20 / 20	1 / 1	0/0	6 / 6	52 / 50
**1,450**	**wet**	4 / 4	0/0	20 / 20	1 / 1	1 / 1	4 / 4	30 / 30
**2,200**	**wet**	0/0	0/0	0/0	0/0	0/0	4 / 4	4 / 4
	**Total**			**628 / 595**			**106 / 105**	**734 / 700**

Supplementary Table S5. Summary of average trait values of butterfly and sphingid species on Mount Cameroon.

	**Proboscis length (mm)**		**Forewing length (mm)**	
	mean	***SD***	***mean***	***SD***
**papilionids**	26.01	6.95	54.95	8.12
**hesperiids**	20.30	10.76	18.60	3.62
**sphingids**	53.24	34.73	33.44	12.99

Supplementary Table S6. Results of linear models analysing effects of elevation on proboscis and forewing lengths of all butterflies and sphingids captured on Mount Cameroon.

	Proboscis length				Forewing length			
	df	*F*	*p*	R^2adj	*df*	*F*	*p*	R^2adj
**all taxa**	6	1.324	0.247	0.030	6	0.847	0.535	0.004
**sphingids**	6	0.626	0.709	0.052	6	1.130	0.356	0.088
**hesperiids**	6	0.720	0.634	0.033	6	0.925	0.480	0.039
**papilionids**	6	0.226	0.966	0.028	6	0.553	0.765	0.065

Supplementary Table S7. Comparison of linear mixed-effect models (with family as random-effect variable) analysing effects of season, elevation, and their interaction on proboscis and forewing lengths of butterflies and sphingids visiting flowers on Mount Cameroon. Models with *ΔAICc* ≤ 2 from the most plausible model were considered as comparable.

	residual df	*residual deviance*	*ΔAICc*	*weight*	*R^2adj*
**Proboscis length**					
season	47	64.062	6.5	0.04	0
**elevation**	**46**	**53.372**	**0**	**0.96**	**0.124**
season × elevation	43	52.951	11.6	0	0.119
**Forewing length**					
season	48	13.718	8.4	0.02	0
**elevation**	**47**	**-0.486**	**0**	**0.98**	**0.046**
season × elevation	44	-4.651	11.7	0	0.119

Supplementary Table S8. Intercorrelation matrix of the measured traits of flower-visiting butterflies and sphingids on Mount Cameroon. After application of Bonferroni correction (39 analyses), only p < 0.001 were considered significant; these are highlighted by **bold**. All correlations were positive.

	Weight	Proboscis length	Forewing length	Forewing width	Body length	Body width	Foreleg length	Midleg length
**Proboscis length**	R^2 = 0.46, p = 0.007							
**Forewing length**	**R^2 = 0.89, p < 0.001**	R^2 = 0.42, p = 0.014						
**Forewing width**	**R^2 = 0.74, p < 0.001**	R^2 = 0.36, p = 0.037	**R^2 = 0.93, p < 0.001**					
**Body length**	**R^2 = 0.97, p < 0.001**	**R^2 = 0.56, p = 0.001**	**R^2 = 0.88, p < 0.001**	**R^2 = 0.74, p < 0.001**				
**Body width**	**R^2 = 0.96, p < 0.001**	R^2 = 0.49, p = 0.004	**R^2 = 0.84, p < 0.001**	**R^2 = 0.68, p < 0.001**	**R^2 = 0.98, p < 0.001**			
**Foreleg length**	**R^2 = 0.89, p < 0.001**	**R^2 = 0.58, p < 0.001**	**R^2 = 0.94, p < 0.001**	**R^2 = 0.87, p < 0.001**	**R^2 = 0.92, p < 0.001**	**R^2 = 0.86, p < 0.001**		
**Midleg length**	**R^2 = 0.90, p < 0.001**	R^2 = 0.52, p = 0.002	**R^2 = 0.94, p < 0.001**	**R^2 = 0.83, p < 0.001**	**R^2 = 0.92, p < 0.001**	**R^2 = 0.89, p < 0.001**	**R^2 = 0.92, p < 0.001**	
**Hindleg length**	**R^2 = 0.94, p < 0.001**	R^2 = 0.41, p = 0.019	**R^2 = 0.92, p < 0.001**	**R^2 = 0.82, p < 0.001**	**R^2 = 0.93, p < 0.001**	**R^2 = 0.88, p < 0.001**	**R^2 = 0.93, p < 0.001**	**R^2 = 0.88, p < 0.001**
